# A Liquid Membrane as a Barrier Membrane for Guided Bone Regeneration

**DOI:** 10.5402/2011/468282

**Published:** 2011-06-12

**Authors:** Daesung Kim, Taeheon Kang, Daniel Gober, Chad Orlich

**Affiliations:** ^1^College of Dentistry, Ewha Womans University, Seoul 158-710, Republic of Korea; ^2^Department of Periodontics, Nova Southeastern University, FL 33328, USA; ^3^Private Practice, Austin, TX 78665, USA

## Abstract

Membranes made of several different materials are available in the market, nonresorbable (e.g. ePTFE), resorbable (e.g. synthetic or collagen) and liguid applicable (e.g. Polyethylene glycol or Atrisorb). The purpose of the present study was to evaluate whether or not in situ application of Atrisorb could be used as a barrier membrane for guided bone regeneration. Ten patients with insufficient alveolar ridge width for implant placement participated in the study. Atrisorb in conjunction with various bone grafts was used to treat 10 different sites, 3 sites treated prior to implant placement and 7 sites in conjunction with implant placement. Augmented sites were allowed to heal for 3 to 7 months, with mean healing time of 4.7 months. Healing was uneventful with no major complications. Two sites experienced a flap dehiscence accompanied by barrier exposure during the initial healing period. Secondary healing was achieved soon after with no signs of infection, giving Atrisorb a barrier exposure rate of 20% for the present study, which corresponds to favorably to that of resorbable membranes. The liquid membrane has the potential of being a viable alternative to traditional resorbable membranes for use in GBR procedures.

## 1. Introduction

Several techniques have been suggested for the regeneration of a deficient alveolar ridge segment. Traditionally, guided bone regeneration (GBR) derives its principles from guided tissue regeneration (GTR). Epithelial and gingival connective tissue cell exclusion, biocompatibility, adequate blood supply, space maintenance, wound stability, and ease of use of a barrier membrane are required for predictable tissue regeneration [1]. Space maintenance is harder to obtain in staged GBR procedures than GTR procedures. GTR relies on the remaining bony walls and teeth to help maintain space for cellular ingrowth and regeneration. In GBR procedures, however, there is a lack of direct support from the surrounding tissues. Therefore, the outcome of GBR depends more on membrane stability, primary flap closure, and postoperative compliance [2].

Many different types of barrier membranes are available on the market today. These are nonresorbable (e.g., ePTFE), resorbable (e.g., synthetic or collagen), and liquid applicable (e.g., polyethylene glycol or Atrisorb). Reports in the literature have proven the efficacy of both resorbable and nonresorbable membranes to exclude soft tissue cells from invading a grafted defect and promote substantial bone regeneration [3–5]. Unfortunately, there are a number of complications that have been reported with the use of these traditional membranes for regenerative procedures, (e.g., exposure, infection, and collapse), especially with nonresorbable membranes [6, 7]. Sometimes, these complications cause failure of the regenerative procedure [8, 9].

Atrisorb (DL-lactide polymer, Atrix Laboratories Inc., Fort Collins, Colo USA) has been used as a barrier membrane, and proven successful for periodontal regeneration [10–12]. Since GBR is based on the principals of GTR, it is reasonable to suggest that Atrisorb can function as a barrier membrane for guided bone regeneration procedures as well. But because it is dispensed in a liquid form, it may be more challenging than traditional membranes to handle during regenerative procedures.

The purpose of this study was to determine the potential of an in situ application technique of Atrisorb to be used as a barrier membrane for guided bone regeneration in achieving adequate horizontal bone regeneration for prosthetically driven implant placement. This study also aimed to evaluate the ease of use and technique sensitivity of a liquid membrane compared to a traditional membrane.

## 2. Materials and Methods

Data were retrospectively collected from ten consecutive patients with insufficient alveolar ridge width for implant placement (no permission from institutional review board human studies committee required). Patients were in good health and had no contraindications to surgical therapy. All patients underwent a complete oral exam and formulation of a comprehensive treatment plan prior to surgery. Diagnostic wax-ups and surgical stents were used as needed to plan ridge augmentation for future prosthetically driven restorations. Presurgical preparation included extensive oral hygiene instructions and treatment to eliminate active periodontal disease, if necessary. 

**Figure 1:**
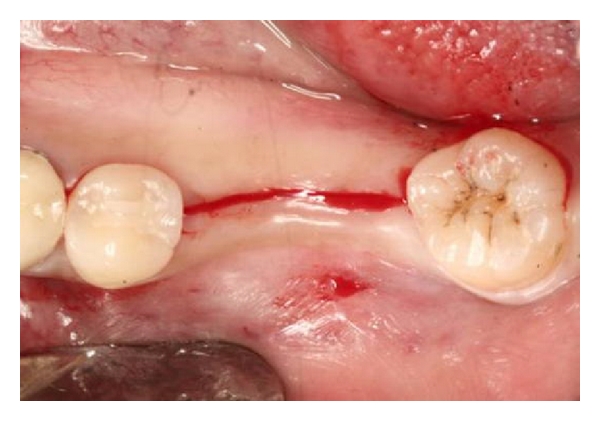
Paracrestal incision edentulous sites number 30 and number 31 for access to alveolar bone.

**Figure 2:**
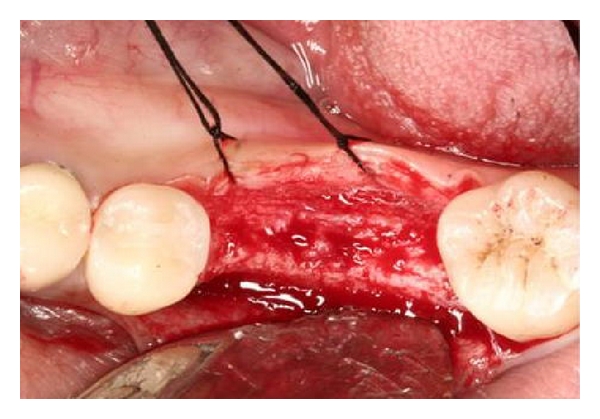
Full-thickness flaps are reflected. Buccal ridge defect is evident.

**Figure 3:**
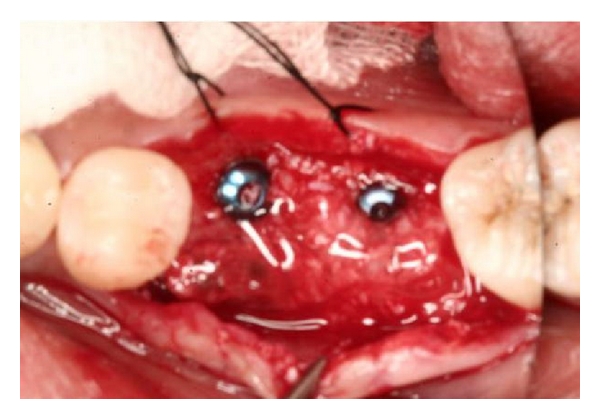
Following placement of implants, bone graft material is placed and Atrisorb liquid is easily applied.

Prior to surgery patients were given 2 grams of amoxicillin and rinsed with a 0.1% aqueous solution of chlorhexidine. All surgical procedures were performed as outpatient procedures under local anesthesia (lidocaine with 1 : 100,000 epi). Atrisorb in conjunction with various bone grafts was used to treat 10 different sites, 3 sites treated prior to implant placement and 7 sites in conjunction with implant placement (Table 1). All surgical procedures consisted of full thickness flaps, with paracrestal incisions made towards the lingual aspect of the ridge in keratinized gingiva (Figures 1 and 2). Vertical releasing incisions were placed providing the flap with a large base and allowing access to the defect. Following flap reflection, any residual soft tissue was removed with curettes, and sutures were used to laterally position flaps as needed. Defects were assessed for adequate buccal lingual width for prosthetically driven implant placement. If ridge width was adequate for implant placement and primary stabilization, implant placement and grafting was performed (three out of ten cases). If ridge width was inadequate for implant placement and primary stabilization, only bone grafting was performed. In all cases, multiple cortical perforations were made on the buccal and crestal bone with a number 8 round carbide bur under copious irrigation. This was done to provide an increase of blood supply and access of progenitor cells to the regenerative site. Various bone grafting materials were then placed and condensed into the defect. Ridge defects were overfilled to compensate for any shrinkage during graft maturation. The surgical field was isolated from saliva contact, ensuring a hemostatic field. Atrisorb barrier was then applied over the bone graft using an in situ method, making sure to entirely cover the particulate and the margins of the graft (Figure 3). Apical undermining of the flap allowed for primary closure and tension-free adaptation of the flap over the grafted area. Horizontal mattress sutures in combination with single interrupted and continuous sutures were placed to allow tension-free closure of the flap. Patients were not permitted to wear removable prosthesis over the surgical area until complete healing and graft maturation had occurred. Patients (received prescription for one week of antibiotic coverage with) were prescribed amoxicillin 500 mg po bid for one week and appropriate analgesics as needed. Patients were also instructed to rinse with 0.12% chlorhexidine rinse twice a day for two weeks. 

## 3. Results

A total of sixteen implants were placed at the grafted sites. All findings are displayed in Table 1. Average healing time was 4.7 months (range: 3.5 to 7 months) before restorative procedure began. Upon reentry for implant placement, the augmented tissue appeared as mineralized bone tissue and Atrisorb seemed to retain its structural integrity. In all of the sites which were augmented prior to implant placement, the bone volume following regeneration was adequate for implant placement. Remnants of Atrisorb were noted in all sites, proving its substantivity of at least 3 months. Survival rate of implants at the grafted sites is 100%. All implants have been in function for 32 months. There has not been any significant marginal bone loss around the implants in the grafted sites.

**Figure 4:**
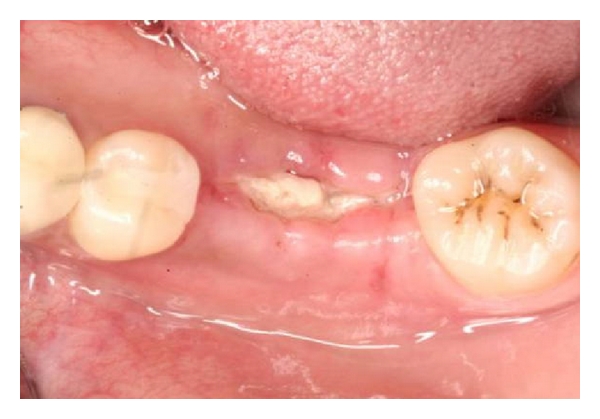
Exposure to Atrisorb at 2 weeks. Notice the lack of epithelialization in the area of exposure.

**Figure 5:**
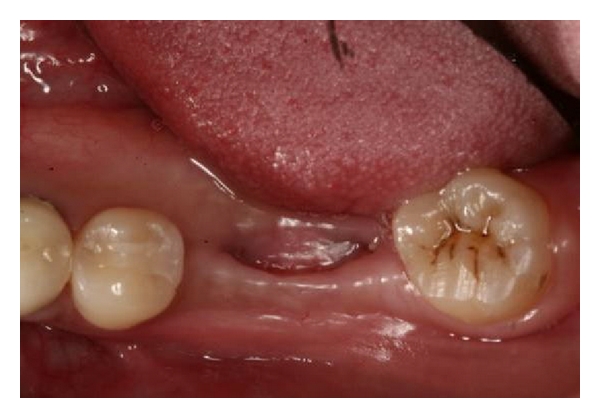
After treatment with chlorhexidine rinse, reepithelialization over the grafted site is evident.

**Figure 6:**
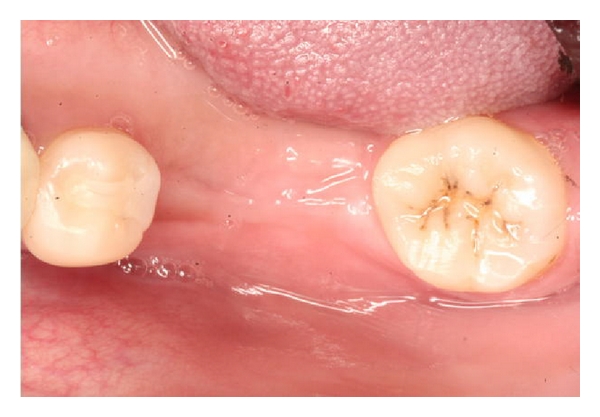
Occlusal view at time of uncovery showing increase in ridge width.

**Figure 7:**
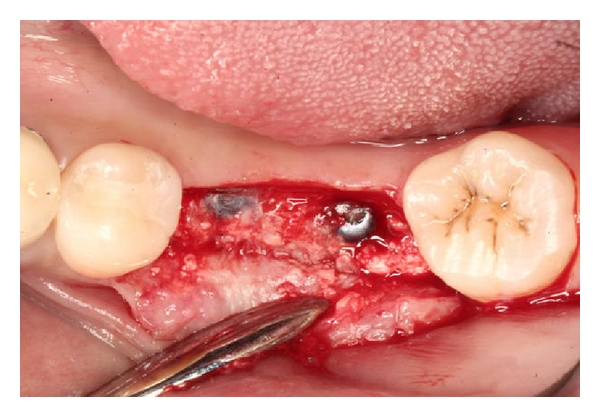
At time of uncovery, Atrisorb material is still intact.

**Figure 8:**
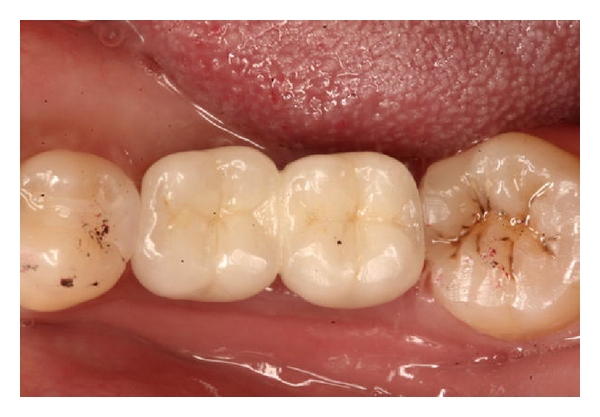
Final restorations in place.

Two sites experienced complications limited to flap dehiscence and exposure of barrier material, resulting in an exposure rate of 20%. One patient (case 5) had a circular shaped exposure on the crest that appeared 2 weeks after surgery (Figure 4). This patient was treated with 0.12% chlorhexidine rinse, and after six weeks there was epithelialization over the exposed barrier (Figure 5), and no further complications or infection was noted (Figures 6, 7, and 8). A second patient (case 10) had a circular shaped crestal exposure at 3 weeks. The patient had some loss of graft material and barrier material during the exposure. The patient was treated with 0.12% chlorhexidine rinse, and epithelialization of the exposed graft site was noted by six weeks. No other complications with healing were noted during the healing process. A minor loss of bone grafts was noticed where there was exposure during the healing phase.

## 4. Discussion

A total of ten sites were treated with a GBR procedure using Atrisorb as a barrier membrane in combination with different grafting materials. The outcome of treatment for all sites was found to be successful. All sites showed regeneration of bone to allow for successful implant placement. This clinical case series demonstrates the potential efficacy of Atrisorb as a barrier membrane for a GBR procedure.

Additionally, in the surgeon's (D. Kim) experience, Atrisorb was easier to handle and more convenient to use than traditional membranes. There was no need to spend time trimming a membrane prior to placement, and there was no concern about the stability of the membrane during flap closure and during the healing period. The Atrisorb liquid was applied quickly and precisely over the bone graft material, and there was no shifting of the membrane during flap closure. 

Substantivity of a membrane is essential for guided bone regeneration. Histological analysis has shown that the kinetics of cell population were greatest during first 2 weeks and subsided by 21 days [13]. Bone maturation continues after the early months following an augmentation surgery [14]. Therefore, in GBR, it is necessary not only to maintain the barrier function for at least 3 weeks, but also to retain its function for at least a few months during the period of bone maturation. In the present study, it was observed that Atrisorb was still present after 3 months.

Another critical factor in regenerative procedures is space maintenance. Even though Atrisorb itself has enough physical strength to maintain its integrity underneath a flap, it cannot be used alone since it does not have sufficient strength to maintain adequate space for regeneration [15]. In order to overcome this limitation, Atrisorb is applied over bone grafting particulate so that it congeals with the particulate and binds to the adjacent bone. This combination results in a material that provides both physical strength and space-maintaining ability. The results of the present study demonstrated that Atrisorb combined with various bone grafts was able to prevent collapse of the grafted site from flap pressure during the healing process and provide adequate space for regeneration of new bone for proper implant placement. 

Although both resorbable and nonresorbable membranes have proven to be predictable options for guided bone regeneration, they have certain limitations. Nonresorbable membranes (e.g., ePTFE) are prone to higher exposure rates [3] and must be removed if exposed [16]. Resorbable membranes (e.g., synthetic or collagen) sometimes suffer from early degradation and an inflammatory response during degradation [17]. Exposure of these membranes can jeopardize the regenerative potential of the grafted site. 

Studies have shown that except for Ossix membranes, most resorbable membranes were not recovered by soft tissue after being exposed [18]. The frequency of soft tissue dehiscence over Atrisorb was comparable to biodegradable membranes and obviously better than nondegradable membranes when used in GBR [3]. Like biodegradable membranes, Atrisorb liquid barrier can experience premature soft tissue dehiscence and exposure, but this does not necessarily jeopardize the regenerative potential of the GBR procedure. In the present study, the two cases with exposure of Atrisorb during healing recovered spontaneously with soft tissue coverage after weeks of irrigation with 0.12% chlorhexidine and oral hygiene. The exposures were not associated with any signs of infection, nor did they cause any significant complications with bone regeneration or interfere with implant placement.

The application of traditional membranes is known to be very technique sensitive. These membranes must be cut and adapted properly to the surgical site to reduce the chance of exposure, and often tacks or sutures are necessary to stabilize the membrane during the healing process, since any shifting of the membrane may compromise its ability to maintain space. Because Atrisorb is a liquid, its application is fast and efficient. There is no need to cut the membrane to fit the grafted area, and no trimming of sharp edges is necessary to reduce chances of exposure. The bioadhesive nature of liquid Atrisorb enables it to adhere directly to the teeth and surrounding bone. This eliminates the need for tacks and stabilizing sutures. In the present study, the Atrisorb barrier did not shift during repositioning and suturing of the flaps. This observation reassured the surgeon that the membrane covered the entire grafted site, maximizing its function as a barrier membrane. It should be noted that isolation of the graft area from blood and saliva prior to application of the Atrisorb barrier is crucial in order to achieve proper covering of the particulate graft and adherence to surrounding structures. The present study demonstrated the ease of use of the Atrisorb liquid membrane in GBR techniques.

## 5. Conclusion

The results of this study indicate that Atrisorb can be used for GBR procedures and that it has the potential to be a viable alternative to traditional nonresorbable and resorbable membranes for use in GBR procedures. In this paper, the in situ application of Atrisorb in conjunction with bone grafts successfully achieved an increase in alveolar ridge width. It was also observed that hard tissue augmentation was not compromised despite the fact that the membrane was exposed to the oral environment for some time. Additionally, the handling of Atrisorb is more convenient when compared to traditional membranes. Future larger scale clinical and histologic studies should be conducted to support the clinical findings presented in this study.

## Figures and Tables

**Table 1:** Intraoperative evaluation, materials, and outcomes.

Case	Gender	Site	Defect	Graft material	Healing (month)	Simultaneous implant placement	Onset of complications after exposure
1	M	7	H	M + BO	5	Y	
2	F	30	H	C + BO	3.5	Y	
3	F	7, 10	H	M + BO	5	Y	
4	F	20	H	C + BO	4	Y	
5	F	29, 30	H	BO	4	Y	2 weeks
6	F	19	H	C + BO	4	N	
7	F	18, 19	H	M	5	Y	
8	F	21	H	M + BO	6	Y	
9	F	13, 14, 15	H	C	3	N	
10	M	29, 30	H	A + C	7	N	3 weeks

H= Horizontal ridge defect.

Graft material: M= MTF(FDB), BO= Bio-Oss, A= Autogenous, C= Cerasorb.
